# Supplementary material for: Identification of photoperiod-induced specific miRNAs in the adrenal glands of Sunite sheep (Ovis aries)
Source: Front Vet Sci. 2022 Jul 22;9:888207. doi: 10.3389/fvets.2022.888207 (PMC9354845; doi:10.3389/fvets.2022.888207)
Supplement: Supplementary file 1 [file Data_Sheet_1.ZIP › Supplementary file/miRDeep2 file.zip!/miRDeep2 file/Know_Structure/SPLP42A1/oar-mir-381.pdf]

miRBase precursor : oar-mir-381
Total read count : 1656
oar-miR-381-5p read count : 4
oar-miR-381-3p read count : 1652
remaining reads : 0

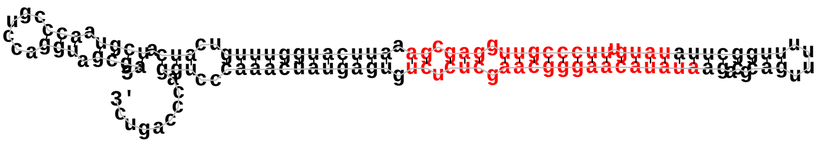

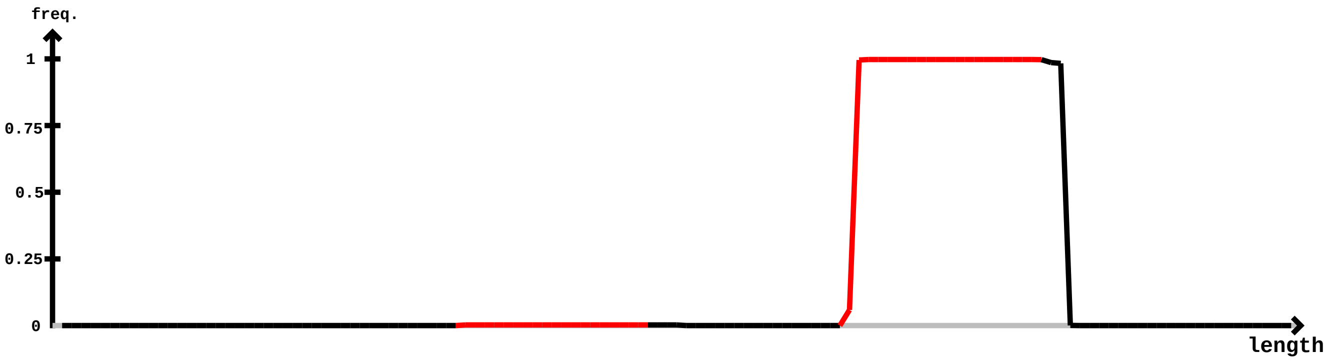


oar-miR-381-3p
oar-miR-381-5p

```
5'- agcgauggaccugcccaaugcuacuacuguuugguacuuaaagcgagguugcccuuuguauauucgguuuuuugacgcagaauauacaagggcaagcucucugugaguaucaaacccuggacccaguc -3' exp
    ((((.(((......))).)))).(((..((((((((((((.((.(((.((((((((.((((((((((((....))).).)))))))))))))))).))).)).))))))))))))..)))........ reads mm
    .........................................agcgagguugcccuuuguauauu................................................................ 4 0
    .................................................................................auauacaagggcaagcucuc........................... 1 0
    .................................................................................auauacaagggcaagcucucu.......................... 10 0
    .................................................................................auauacaagggcaagcucucugu........................ 86 0
    ..................................................................................uauacaagggcaagcucucu.......................... 8 0
    ..................................................................................uauacaagggcaagcucucug......................... 5 0
    ..................................................................................uauacaagggcaagcucucugu........................ 1538 0
    ..................................................................................uauacaagggcaagcucucugug....................... 1 0
    ...................................................................................auacaagggcaagcucucugu........................ 3 0
```
